# Supplementary material for: Effects of Marquandomyces marquandii SGSF043 on the Germination Activity of Chinese Cabbage Seeds: Evidence from Phenotypic Indicators, Stress Resistance Indicators, Hormones and Functional Genes
Source: Plants (Basel). 2024 Dec 27;14(1):58. doi: 10.3390/plants14010058 (PMC11722606; doi:10.3390/plants14010058)
Supplement: Supplementary file 1 [file plants-14-00058-s001.zip › plants-3261234-supplementary.pdf]

Appendix

Supplementary data

Table S1 Effects of 96h *M. marquandii* SGSF043 on germination growth of Chinese cabbage seeds

Treatment	The number of viable fungi(mL)	Shoot height (cm)	The young buds grow(cm)	Stem diameter (cm)	Number of secondary roots (article)	Root-shoot ratio (Dry weight ratio)
M.marquandii SGSF043	LM(1×10^6)	36.63±0.09c	16.51±0.01c	1.37±0.13b	11.03±0.53c	0.24±0.02a
	MM(1×10^7)	43.64±0.07ab	17.70±0.02b	1.54±0.15a	11.67±0.92b	0.23±0.01a
	HM(1×10^8)	46.06±0.1a	20.82±0.01a	1.58±0.22a	13.57±0.37a	0.25±0.03a
Control（PDB）	—	42.94±0.06b	14.81±0.03d	1.11±0.12c	8.83±0.65c	0.11±0.01b

Note: The results are mean ± standard deviation for 30 replicates. Different lowercase letters in the same column indicate differences between treatment groups ($P<0.05$), n=30.

Table S2 Primer sequences used for qRT-PCR validation

Genes	Forward primer（5'-3'）	Reverse primer（5'-3'）
Br18s	CTCAGTCCAAAAGAGGTATTCT	GTAGAATGTGTGATGCCAGATC
SGSF043	GCCTGTTCGAGCGTCATTTC	TGGGGTTGTTTTACGGCAGT
BrPAO	CGAAGCTCACGTTCACTCCA	GCTGACTCTTGCCATACCGT
BrSAG12	GACCCTCATCGTCCGAATCC	TAGGTTGGACAGGCAGCTTG
ARF7	GCAACCACCTCAAATGCTGGTGAG	GGTGGAAGGCGACGTTGAAGAGG
ARF19	CCCGTTATAGCGGCTATGATGAAC	ATATCGTTTTCGTGATCGGTGTAG
BnNCED3	GTGGAAGTCGGAGTTACAGATAG	CCAAGTCACTAGCTCCGATAAA
BrPOD	CTTAGTCGCGGCAGGGGAAT	TCGACCTCGCAAACTGAGCA
BrSOD	GCACCCGAGGATGCTAATCG	TACCACAAGCAACACGGCCT
BrCAT	GTTTGATCCTGTCCGGTGCG	CTCACGTTCAGACGGCTTGC
IAA	CGTCTCCTCACTCATCGCTC	TTCGTCTTTGGGTGAGTCGG
MYC3	ATCTTGAAGCGTCGGTGGTT	TGCCTCTCTGCTTCCACATG
